# Prevalence and factors influencing depression among empty nesters in China: A meta-analysis

**DOI:** 10.1186/s12877-023-04064-0

**Published:** 2023-05-30

**Authors:** Caini Song, Libo Yao, Huisu Chen, Ying Song, Lihua Liu

**Affiliations:** 1 Department of Nursing, College of Medicine, Hunan Normal University, Hunan Province, China; 2 Minimally Invasive Surgery Center of The First Hospital of Changsha, Hunan Province, China; 3 Department of Respiratory Medicine, Hunan Provincial People’s Hospital, Hunan Province, China

**Keywords:** Depression, Empty-nesters, Influencing factors, Meta-analysis

## Abstract

**Background:**

Empty nesters are older people who live alone or an older couple without children to care for them. In China, empty nesters make up a significant community and are more likely to experience emotional issues, particularly depression. This study investigated the prevalence of depression and the factors influencing depression among Chinese home-bound empty nesters using meta-analysis.

**Methods:**

Based on previous studies, we used search terms relating to empty nesters and depression in English and Chinese. Databases, including China Journal Full Text Database (CNKI), Wanfang, Wipu, China Biomedical Literature Database (CBM), PubMed, Web of Science, Embase, The Cochrane Library, and UptoDate, were searched in April 2022, for relevant articles. Details including names of authors, year of publication, region of investigation, study type, sample size, depression detection scale, depression detection rate, and influencing factors were captured. The heterogeneity of the studies was assessed based on the I^2^ index, and data analysis was performed using Stata 16.0 software.

**Results:**

A total of ten research articles involving 5337 Chinese empty nesters were evaluated in the present meta-analysis. The overall prevalence of depression among empty nesters in China was 43%. The prevalence of depression among urban empty nesters was 38% (95% CI: 0.24,0.52), and 36% (95% CI: 0.18,0.55) among rural empty nesters. Many factors, including female, income, marital status, chronic illness, relationship with children, and social support were linked to depression among urban empty nesters.

**Conclusion:**

The prevalence of depression among empty nesters was 43%. Therefore, based on the factors influencing depression, government departments can intervene early to improve the mental health of empty nesters.

**Limitations:**

The meta-analysis only included cross-sectional studies. Therefore, there is a need for more future original studies investigating depression among empty nesters in China.

## Background

Empty nesters are older people who live alone or an older couple without children to care for them [1]. According to data from the seventh census [2], there were 264 million individuals aged over 60 years in China, accounting for 18.7% of the China’s population. At the same time, there were 190 million individuals aged over 65 years, accounting for 13.5% of the population.

According to the survey, the size of China’s empty nesters will be nearly 150 million in 2020, with 7.72 million senior citizens living alone in the empty nesters [3]. Previous researchers have found that depression is significantly higher among empty nesters than non-empty nesters. 79.7% of empty nesters in China were depressed, compared to 65.8% of non empty nesters [4]. The prevalence of mild and moderate depression among empty nesters in China was 72.3% and 7.4%, respectively. Su et al. [5] found that the prevalence of depressive symptoms among empty nesters was 73.3%. Among them, 63.4% were mild depression, whereas 9.9% were moderate to severe depression. Zhang et al. [6] showed that the prevalence of depression among empty nesters in China was 64.2%.

Empty nesters are more likely to suffer from physical and psychological problems [7], such as functional impairment, low quality of life, high risk of death, and depression, due to the long-term lack of companionship and emotional support from children. If not effectively treated, depression leads to sleep disturbances, suicidal thoughts, and participation in high-risk behaviors [8]. According to research, adequate social support improves the psychological well-being of empty nesters [9]. Other research has found that empty nesters are more likely to experience anxiety and loneliness, increasing their risk of mental health problems [10]. The risk of psychological conditions among empty nesters is higher than physical health conditions, which may be related to various stressors, such as the absence of children and a lack of hobbies and social support.

According to a previous meta-analysis, depression among empty nesters in China was as high as 38.6% [11]. Therefore, empty nesters are a high-risk group for depression. In addition, there are differences in the onset of depression among empty nesters with different characteristics. However, the above study did not investigate the factors influencing the prevalence of depression among empty nesters in China.

This published meta-analysis retrieved a total of 7 databases, and articles published before September 2021. It has some limitations, such as noncomprehensive search databases, inclusion of outdated articles, and inclusion of low quality literature. Therefore, this meta-analysis aimed to address these shortcomings and investigate the prevalence and factors influencing depression among Chinese home-bound empty nesters.

## Methods

This systematic review was evaluated using the PRISMA (Preferred Reporting Items for Systematic Evaluation and Meta-Analysis) 2020 statement [12], and it is now registered in the PROSPERO database (registration number: CRD42022330177). A total of nine databases, six of which publish articles written in English, were searched for relevant studies published since the inception of databases to April 2022. All databases had a literature quality score of ≥ 7, indicative of moderate quality and higher credibility.

### Search strategy

We conducted systematic search in PubMed, Web of Science, Embase, The Cochrane Library, UpToDate, CNKI, Wanfang, Wipe, and CBM for articles published since inception of the databases to 20 April 2022. Extra studies were extracted from reference section of the relevant articles.

Based on previous studies [11], the Chinese search terms were (“kong chao lao ren”) AND ((“yi yu”) OR (“yi yu zheng zhuang”)) AND ((“ying xiang yin su”) OR (“wei xian yin su”) OR (“xiang guan yin su”)), whereas the English search terms were ((“empty nesters”) AND (“depression”)) AND (((“influencing factors”) OR (“risk factors”)) OR (“associated factors”))).

### Inclusion and exclusion criteria

Inclusion criteria: (1) Literature on the prevalence and factors influencing depression among home-bound empty nesters living within China published in Chinese and foreign databases; (2) the study population was older people aged 60 years or older; (3) the study type was a cross-sectional study; (4) the publication language was Chinese or English.

Exclusion criteria: (1) the study site was a hospital or nursing home; (2) incomplete or inaccessible data; (3) duplicate publications; (4) review and conference-type literature; (5) poor quality score of the literature.

### Data extraction

Two researchers screened the literature simultaneously and independently, extracted and cross-checked the data. Disagreements were arbitrated by a third party. The following information was extracted from the data: the year of publication, the first author, the region of investigation, the study type, the sample size, the depression detection scale, the depression detection rate, and the influencing factors. Corresponding authors were contacted to obtain missing data. The article was rejected if the author could not be reached or provide data.

### Literary criticism evaluation method

The quality of the included literature was assessed by two institutes using the cross-sectional study quality assessment criteria recommended by the American Agency for Health Care Quality and Research (AHQR) [13]. There were 11 items, and “Yes” was counted as 1 mark, whereas “No” or “Unclear” was counted as 0 marks. The higher the total scores, the higher the quality of the literature, where ≥ 8 was high quality, 6–7 was moderate, and ≤ 5 was low quality. Studies with scores below 7 were not included in the final analysis.

### Statistical analysis

The data were analyzed using Stata 16.0 software. The prevalence of depression was calculated by extracting the total sample size and the number of cases from the included study. For meta-analysis of risk factors, results of univariate analysis (when multivariate analysis results were not reported) or multivariate logistic regression analysis were extracted from the included studies. In addition, for the results of multiple linear regression analysis, we calculated the correlation coefficient between research factors and depression (continuous variable) based on the method in the previous paper [14]. Then, we converted the correlation coefficient into an odds ratio for analysis (https://www.psychometrica.de/effect_size.html. Psychometrica. DOI: 10.13140/RG.2.2.17823.92329).

The effect sizes were described using OR values and 95% CIs, and forest plots were drawn. Heterogeneity was assessed using I^2^ and P values. A fixed-effects model was used if homogeneity was good (I^2^<50%, P ≥ 0.1). On the other hand, if heterogeneity was strong (I^2^ ≥ 50%, P<0.1), a random-effects model was used.

## Results

### Literature search

**Fig. 1:**
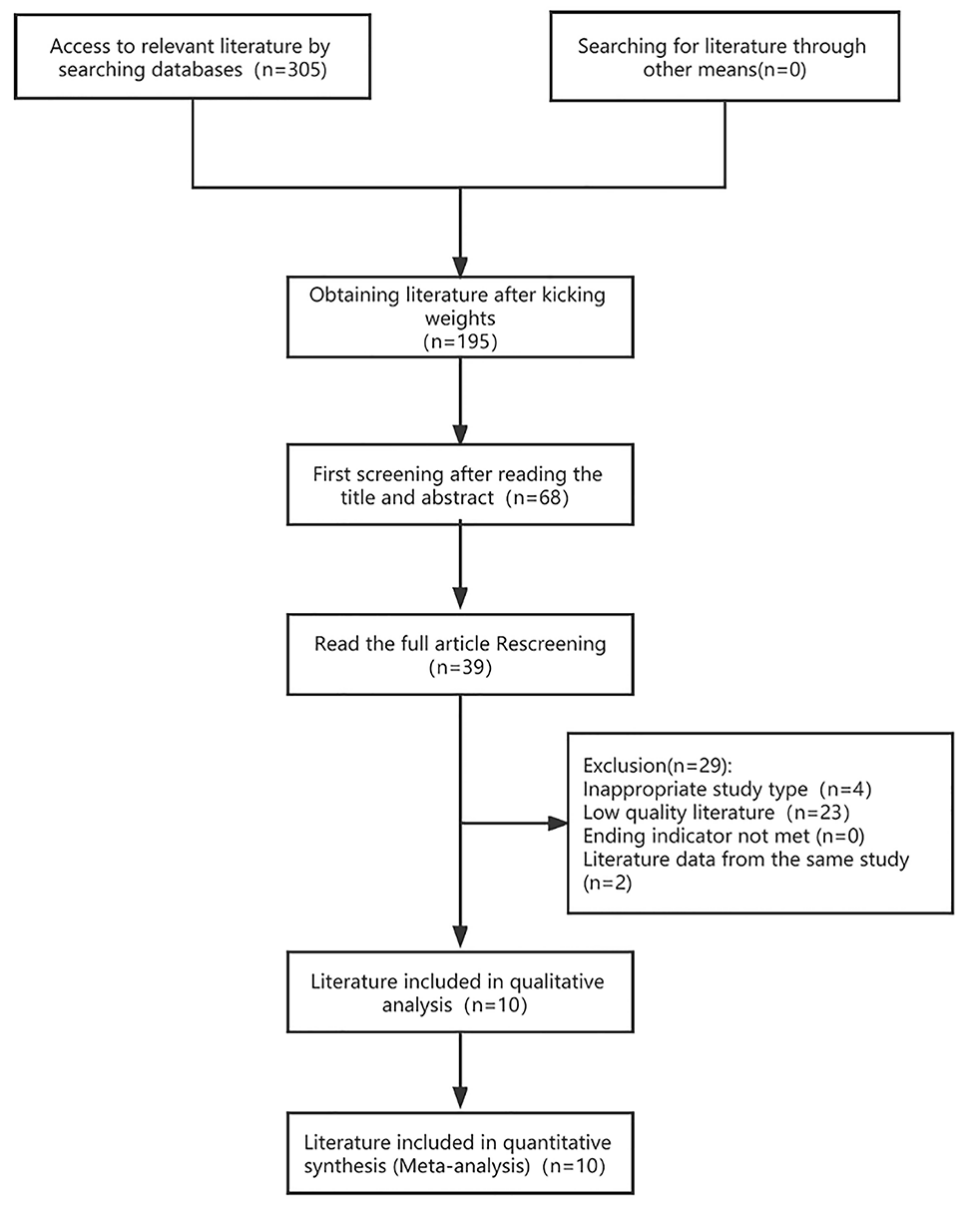
Study selection flowchart

A total of 305 papers were obtained in the database search, and 0 were obtained through other sources. First,110 papers were excluded since they were duplicates, leaving 195 papers. Second, 127 papers were excluded based on their titles and abstracts since they were conducted on patients in inpatient or nursing facilities, leaving 68. Then, 39 articles were obtained after reading the full text for re-screening. Finally, based on the quality score of the literature and the inclusion and exclusion criteria, 10 articles were retained. Figure 1 depicts the detailed process of identifying eligible literature.

### Evaluation of study characteristics and methodological quality

Table 1 shows the basic characteristics of the ten articles. All of the articles were cross-sectional studies. Nine of the ten articles were published in Chinese, with one published in English. Furthermore, the sample size for each article ranged from 187 to 2911. This meta-analysis analyzed 5337 Chinese empty nesters. Seven of the ten articles assessed the depressive symptoms using the Geriatric Depression Scale (GDS), whereas two assessed the depressive symptoms using the Self Scale of Depression (SDS). At the same time, one article assessed the depressive symptoms using the center for the epidemiological survey (CED-S). Regarding the quality of the literature, all cross-sectional studies scored between 7 and 9, with 8 being moderate quality and 2 being high quality.

**Table 1:** The fundamental characteristics of the ten included studies

No.	First author	Year	Publication language	Sample size	Prevalence	Measure tool	Study quality score
1	Yu Yan [15]	2022	Chinese	2911	36.40%	CES-D	8
2	Jiangang Tan [16]	2021	Chinese	992	17.14%	GDS	7
3	Xiaoli Zhong [17]	2021	Chinese	463	56.80%	GDS	7
4	Zhifang Shen [18]	2012	Chinese	1785	19.80%	GDS	7
5	Changkuan Jia [19]	2007	Chinese	328	23.80%	GDS	7
6	Shoumei Jia [20]	2007	Chinese	229	15.30%	GDS	7
7	Lin Zhang [21]	2012	Chinese	2790	44.30%	SDS	9
8	Fang Liang [22]	2014	Chinese	187	79.70%	GDS	7
9	Liqin Xie [4]	2009	English	231	79.70%	GDS	7
10	Xue Cao [23]	2012	Chinese	454	55.30%	SDS	7

### The overall prevalence of depression

**Fig. 2:**
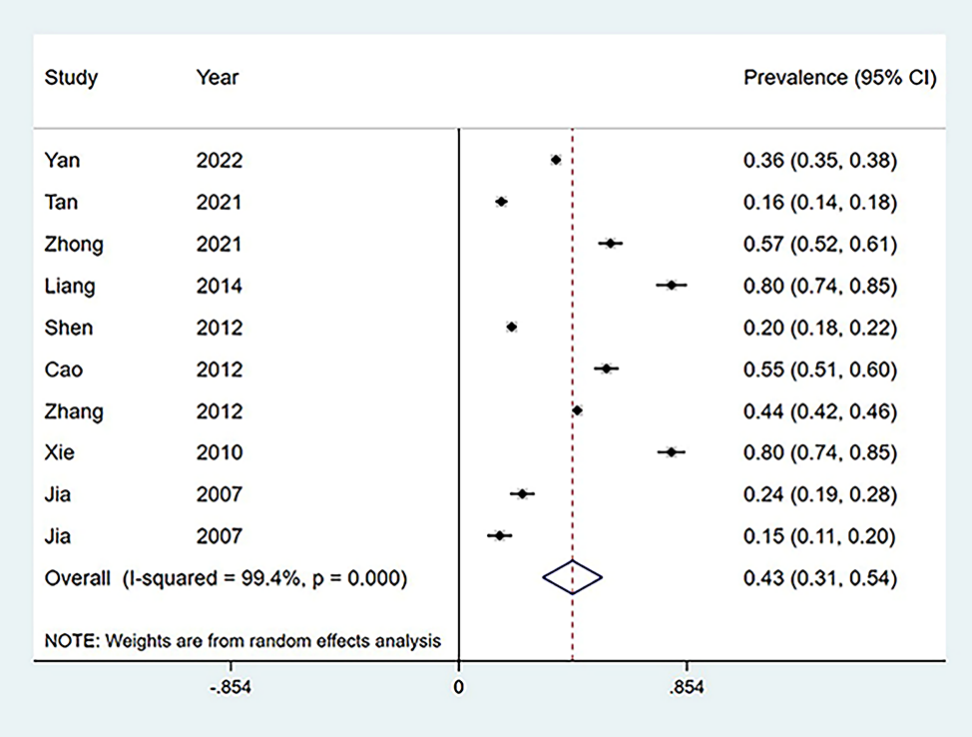
Forest plot of the prevalence of depression among empty nesters in China

**Fig. 3:**
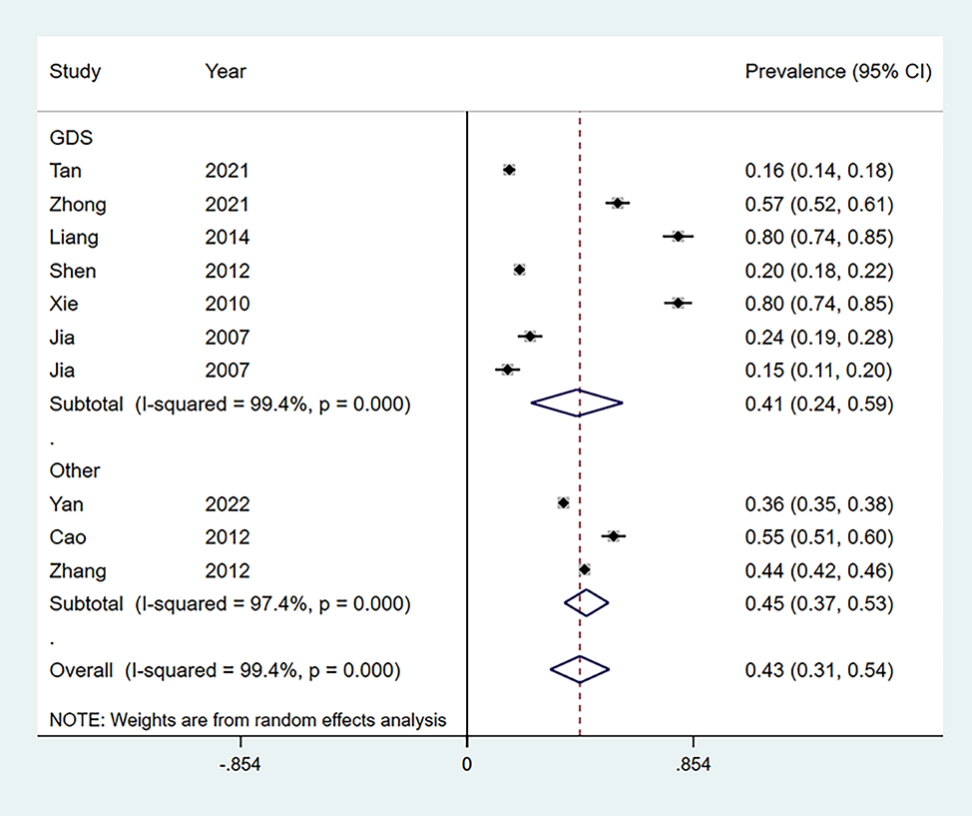
Different scales of forest plots measuring depression prevalence

**Fig. 4:**
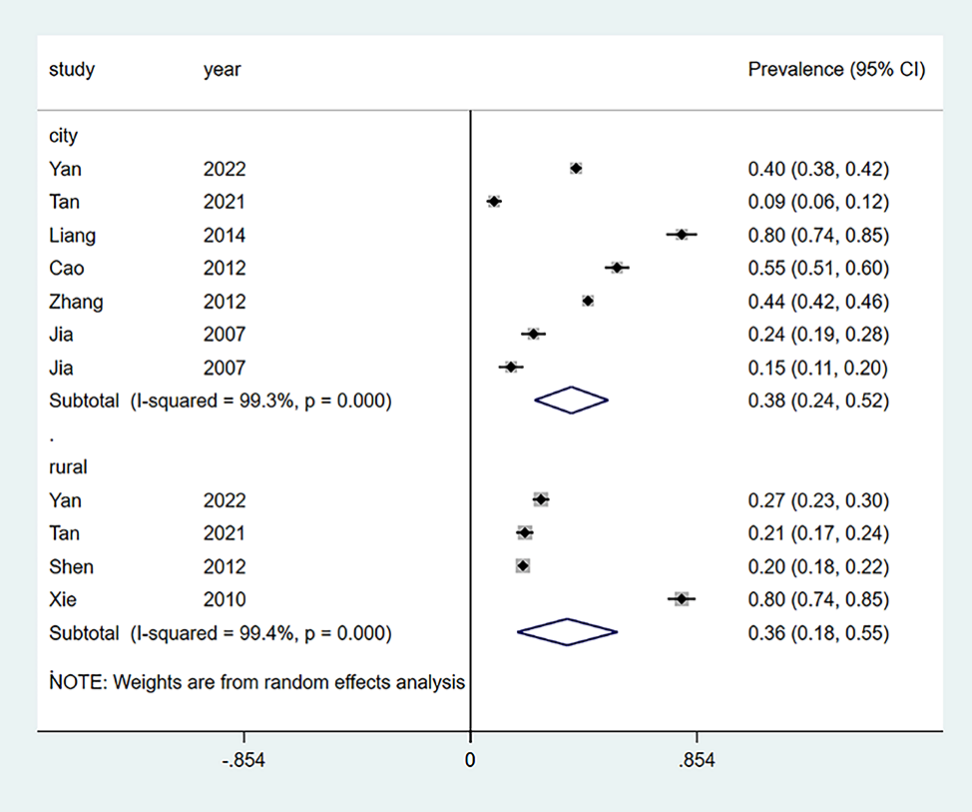
Depression prevalence in various regions is represented as a forest chart

As shown in Fig. 2, the prevalence of depression among Chinese empty-nesters in each study was calculated, which ranged from 15 to 80%. The overall prevalence of depression for the ten studies [4, 15–23] was 43% (95% CI: 0.31,0.54) (Fig. 2). Depending on the scale, the detection rate was 41% (95% CI: 0.24,0.59) for the GDS scale [4, 16–20, 22] and 43% (95% CI: 0.31,0.54) for the other scales [15, 21, 23] (Fig. 3). Depending on the region, the detection rate of depression was 36% (95% CI: 0.18,0.55) in rural empty nesters [15, 16, 18, 4] and 38% (98% CI: 0.24,0.52) in urban areas [15, 16, 19–23] (Fig. 4).

### Depression-related factors

**Fig. 5:**
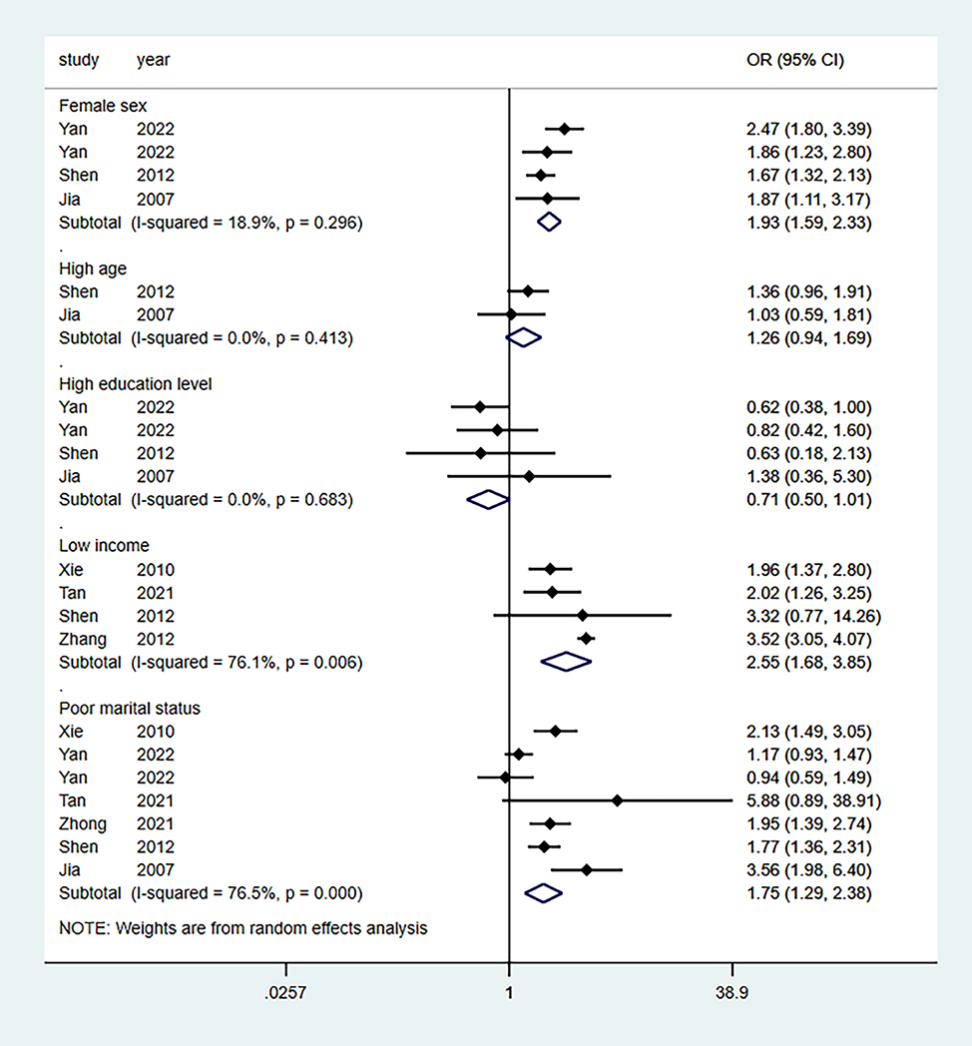
Forest plots of common demographic and socioeconomic factors (age, sex, education, income, and marital status) for depression among empty nesters in China

**Fig. 6:**
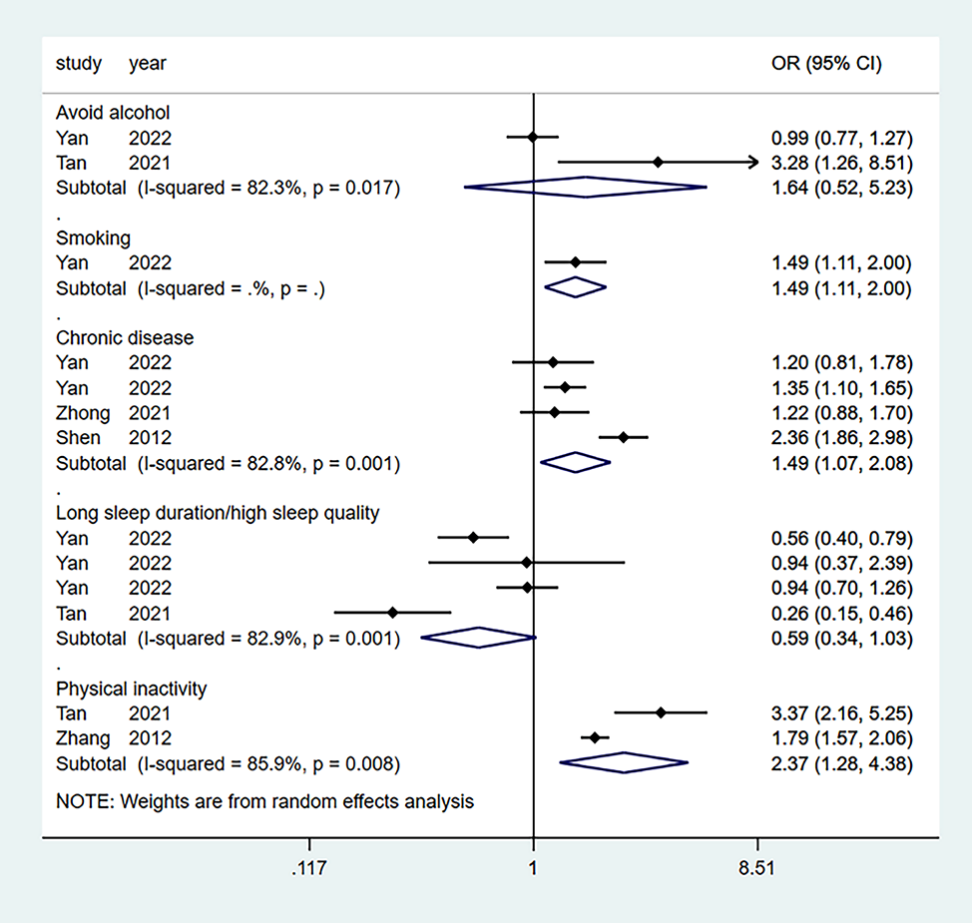
Forest plots of lifestyle-related factors (alcohol, smoking, sleep duration or quality, and physical inactivity) and chronic disease for depression among empty nesters in China

**Fig. 7:**
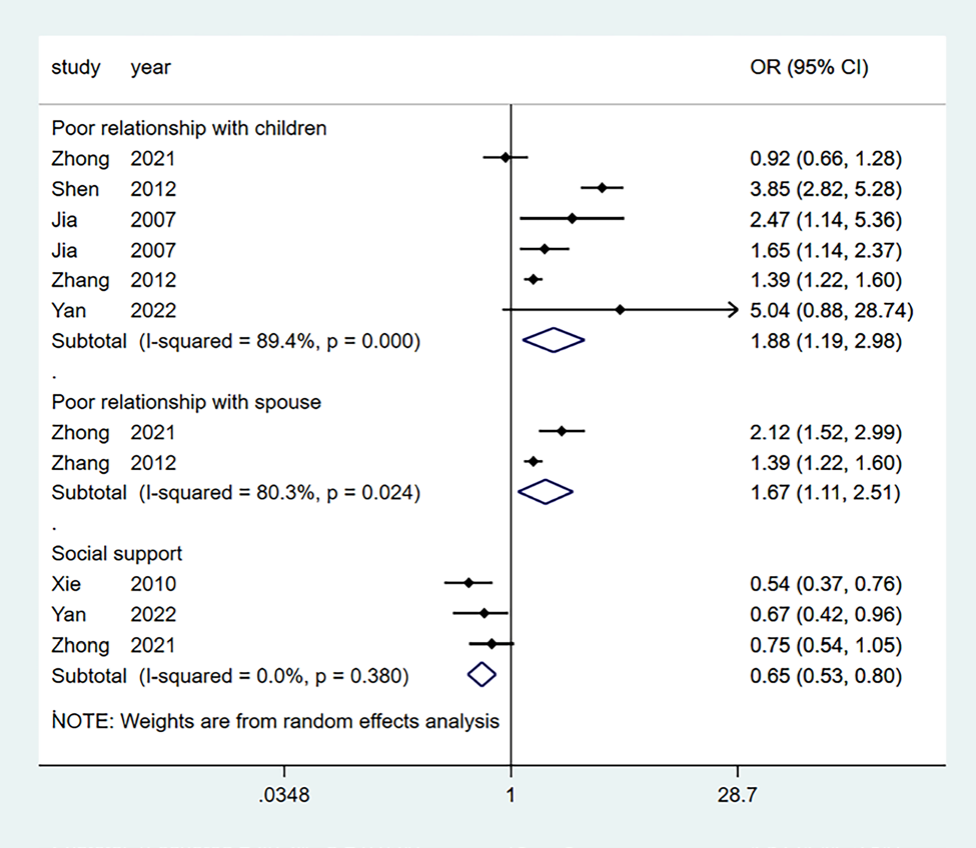
Forest plots of social relation related factors (poor relationship with children, poor relationship with spouse, and social support) for depression among empty nesters in China

Seven studies aggregated the effect of marital status, and six aggregated the effects of a relationship with children. Four studies aggregated the effect of females, income, and chronic illness. At the same time, three studies aggregated the effect of social support. Furthermore, four effect factors (factor age, education level, alcohol, sleep) were not statistically significant, while the other three (factor smoking, physical inactivity, poor relationship with spouse) were only available in one or two studies. Table 2 lists the 13 influencing factors considered in the meta-analysis. Figures 5, 6 and 7 show forest plots of the influencing factors. Six factors were statistically significant after combining the ORs: Low income (OR = 2.55; 95% CI: 1.68–3.85), marital status (OR = 1.75; 95% CI: 1.29–2.38), chronic illness (OR = 1.49; 95% CI: 1.07–2.08), poor relationship with children (OR = 1.88; 95% CI: 1.19–2.98), and social support (OR = 0.65; 95% CI: 0.53–0.80).

Notably, only two articles mentioned additional influencing factors in addition to those listed above, such as physical pain, daily living ability, and self-assessment of health status. There is a need for more research to confirm the link between these variables and depression.

**Table 2:** Risk Factors Meta-Analysis

Factors	No. studies	Sample size	I^2^ (%)	Pooled OR	95% CI		Test for overall effect (P value)
					Lower	Upper	
Female sex	4	4696	18.9	1.93	1.59	2.33	0.296
High age	2	2113	0.0	1.26	0.94	1.69	0.413
High education level	4	5024	0.0	0.71	0.50	1.01	0.683
Low income	4	5798	76.1	2.55	1.68	3.85	0.006
Poor marital status	7	6730	76.5	1.75	1.29	2.38	0.000
Avoid alcohol	2	3903	82.3	1.64	0.52	5.23	0.017
Smoking	1	2911	0	1.49	1.11	2.00	0.000
Chronic disease	4	5159	82.8	1.49	1.07	2.08	0.001
Long sleep duration/high sleep quality	4	3903	82.9	0.59	0.34	1.03	0.001
Physical inactivity	2	3782	85.9	2.37	1.28	4.38	0.008
Poor relationship with children	6	8506	89.4	1.88	1.19	2.98	0.000
Poor relationship with spouse	2	742	80.3	1.67	1.11	2.51	0.024
Social support	3	3395	0.0	0.65	0.53	0.80	0.380

## Discussion

To the best of our knowledge, this is the first meta-analysis of English and Chinese literature to examine the prevalence and influencing factors of depression among empty nesters in China. This meta-analysis completes the current research system and provides a reference for subsequent studies. According to the meta-analysis findings, the overall prevalence of depression among empty nesters in China was 43% (95% CI 0.31 to 0.54 for the overall pooled estimate). Based on our findings, nearly half of the Chinese empty nesters are depressed. However, the prevalence of depression differed by region.

For example, a study on depression among empty nesters in Hangzhou [16], found that 17.14% were depressed. Yan et al. [15] surveyed 2911 respondents aged 60 and older from the 2018 China Health and Aging Tracking Survey (CHARLS) and found that the prevalence of depression among urban empty nesters was 26.2% and 39.9% among rural empty nesters. Hossain et al. [24] conducted a cross-sectional study of 30,366 respondents aged over 60 years in India and found that depression in older people was 30.3%. A study of the global prevalence of depression in older people found a prevalence of 31.74% [25]. At this time, the prevalence of depression in other study populations was lower than the results of this study compared to the individual published studies. After investigating the reasons for this, we discovered that empty nesters face more stressful life situations than the general population.

Studies have revealed that empty nesters in China have less optimistic mental health [26]. Studies show that older people have higher psychological disorders and a higher risk of depression [27]. In this study, females were an influential factor in depression among empty nesters. Sileo et al. [28] found a higher prevalence of depression in females than in males in Uganda. Other studies, such as the one conducted in India [29], have found that men have a lower prevalence of depression than women, consistent with our findings. Due to the declining estrogen levels, women in their golden years are more prone to anxiety and depression when faced with loneliness [30].

Furthermore, the findings of this study revealed that empty nesters with lower incomes were at a higher risk of depression, possibly due to financial strain, since they cannot work to earn money to share the financial burden for their children. In addition, they are in poor health and require long-term medication [31]. Financial difficulties are a risk factor for depression. Furthermore, compared to the high-income group, the low-income group cannot satisfy their hobbies and express their negative emotions on time, consistent with previous research [32].

Marital status predicts depression in empty nesters. Our findings suggest that widowed empty nesters are more likely to be depressed than married empty nesters, consistent with previous research [33]. This could be because married empty nesters have spouses and rarely feel lonely [34]. Spouses can provide emotional support when they encounter conflicts, thus alleviating negative emotions. At the same time, the support provided by their spouse plays an important role in their lives, enhancing positivity and reducing depression. In contrast, the absence of a spouse can cause loneliness and emptiness, exacerbating the appearance of depression.

Chronic illness has been linked to depression in empty nesters, consistent with Chinese academic research [35]. According to research, depression is related to poor physical health and chronic illness. Over 50% of those with depressive symptoms had at least one chronic disease. On the other hand, approximately 20% had a combination of at least two chronic diseases [36]. Long-term accumulation caused by chronic illness, the high medical burden on the family, severe guilt in older people due to the high cost of treatment, and severe psychological burden due to the reduced quality of life caused by the torment of illness, may all exacerbate the degree of depression [37].

This study reveals that empty nesters with poor relationships with their children have poorer mental health since China is based on inheritance and family. Parents have high expectations of their children and want feedback when they age. However, the fact is that children have their ideas and want their own private space, and the expectations of older people are not met. Therefore, it aggravates the emergence of depression [38].

Finally, we realized that social support protects against depression among empty nesters. Social support refers to moral or material support and assistance from all aspects of society, including relatives and friends. Consistent with the findings of previous studies [39], higher social support levels protect the mental well-being of older people [40]. Social support is a crucial factor against stress and poor health outcomes [41]. For example, since social support can fight negative emotions, social support from loved ones, friends, and society plays a key role in maintaining psychological well-being. As a result, good social support is crucial for empty nesters. On the other hand, empty nesters are more likely to be depressed.

## Limitations

We recognize that this meta-analysis has limitations. First, this study only included published Chinese and English journal literature by Chinese scholars, limiting the comprehensiveness of the data. Second, the studies included were cross-sectional and contained many uncertain confounding factors. The meta-analysis was significantly heterogeneous. Regional differences, demographic characteristics, the prevalence of underlying diseases, study design, depression diagnosis methods, and statistical analysis methods may have caused this. However, the source of heterogeneity was not observed using subgroup analysis. This could be a result of the few studies that are currently accessible, where subgroup analysis makes it challenging to identify sources of heterogeneity. There is a need for more articles for further study. Despite these limitations, in the present study, the small sample size of a single study lacked representativeness, and differences in the prevalence and influencing factors of depression between regions were eliminated using meta-analysis. In addition, we aimed to provide a more objective picture of the relationship between depression and related factors for empty nesters in China. This study will serve as a resource for policymakers to improve the mental health of empty nesters.

## Conclusions

The findings indicated that the prevalence of depression among empty nesters in China was not encouraging. Six influential factors were summarized, one of which was protective, and the rest were effects. However, there is a need for more high-quality studies and representative samples. Future research should concentrate on improving the mental health of empty nesters, such as depressive symptoms. Relevant authorities in China should formulate policies and measures to improve the mental health of empty nesters.

## Data Availability

Datasets are available through the corresponding author upon reasonable request.

## Abbreviations

PRISMA: Preferred Reporting Items for Systematic Evaluation and Meta-Analysis
AHQR: American Agency for Health Care Quality and Research
GDS: Geriatric Depression Scale
SDS: Self Scale of Depression
CED-S: Center for epidemiological survey
